# A Sensory Update to the C*haîne Opératoire* in Order to Study Skill: Perceptive Categories for Copper-Compositions in Archaeometallurgy

**DOI:** 10.1007/s10816-017-9356-9

**Published:** 2017-12-16

**Authors:** M. H. G. Kuijpers

**Affiliations:** Faculty of Archaeology, Leiden University, Room A105, Einsteinweg 2, 2333 CC Leiden, Netherlands

**Keywords:** Bronze Age, Metalworking, Skill, *Chaîne opératoire*, Perceptive categories, Archaeometallurgy

## Abstract

This paper introduces the methodology of perceptive categories through which an empirical analysis of skill is achievable, taking European Bronze Age metalworking as a case study. Based on scientific data provided by the material sciences, in this case compositional and metallographic analyses of Late Copper Age and Early Bronze Age axes, the thresholds to categorise and interpret these data, and organise them in a *chaîne opératoire*, are centred on the human senses—and thus on metalworking as a craft. This is a pragmatic approach that appreciates scientific measurements of metal objects as essential empirical evidence whilst recognising that a considerable share of these archaeometric data are inapt or too detailed for an understanding of skill. This empirical approach towards skill is relevant to our knowledge of the role of crafts and materials in the past. After all, skill is a fundamental asset for the production of material culture, and a distinct human-material relationship characterised by an intimate form of material engagement.

## Introduction

There are two distinct frameworks in which prehistoric technologies are studied: a material framework and a social framework. The former is universal, the latter contextual. Typically, these frameworks have little to no overlap in terms of methodologies, focus, and understandings (Dobres 2010; Jones 2002; Killick 2004; Thornton 2009). In a recent paper, a third framework was suggested: the psychophysical framework. This framework takes into account prehistoric skill, cognition, and the senses (Kuijpers 2013).

Skills are fundamentally dependent on a sensory reading of the material. Every move craftspeople make, turning their idea into practice, happens *in response* to their material. What is important is that this response occurs on the level of perceivable qualities rather than properties of a material (Frayling 2011; Hurcombe 2007; Pye 1995). The focus of the methodology proposed here aims to explore this sensory dimension, and to use this to construct *perceptive categories* as a means of differentiating the production of prehistoric axes on the basis of their metallographic data.

Perceptive categories emphasise the qualities, behaviour and performance of materials that are important to craftspeople and attempts to associate these with the properties and processes for which scientific measurements are available. The theoretical underpinning of this essential nuance between properties and qualities I explore in detail elsewhere (Kuijpers 2018). Here, I simply recognise craft theory as a valuable approach towards archaeological materials, and aim to show how it may be implemented and how it significantly impacts our understanding of metallurgical data.

In the following, I will work through three main arguments: that skill needs to be incorporated in our archaeological investigations of Bronze Age metalwork, because skills have been given much weight in our conceptualisation of the Bronze Age from Childe (1963) onwards. That to uncover technical skill we need to understand how craftspeople recognise and respond to differences in their material, which implies the use of material sciences and the data produced by this field, but it in a manner that is more attuned to how craftspeople perceive materials. That this is possible because the difference between scientific knowledge and practical knowledge of a craftsperson is a variance in acuity and metaphors rather than an incompatibly dissimilar understanding of reality. These three arguments are subsequently brought together in the idea of *perceptive categories* and implemented as a sensory update to the *chaîne opératoire* of metal production, concentrating on the transformation from raw metal (ingots or scrap) to finished object.

## Metalworking Skill: Appreciated but Uncharted

Skill is considered both cause and effect as well as a signpost for specialists and craft specialisation (*e*.*g*. Apel 2008; Costin 2001; Hruby *et al.* 2007; Olausson 2017; Sofaer 2010). A good example are the interpretations of metalworking in Bronze Age Europe. To support the idea that metalworking is distinct from and more specialised than other crafts, skill and knowledge are often put to the fore (Harding 2000, p. 239; Kuijpers 2012, 2013 with examples). This effectively places the metalworker and metalworking skills at the very core of the prevalent models of social complexity in the Bronze Age (Kristiansen and Larsson 2005; Ottaway and Roberts 2008; Rowlands 1984). This obviously warrants a proper understanding of skill, but when scrutinised, one finds that the perceived skills of prehistoric metalworkers are largely conjectured on the basis of circumstantial theoretical associations and positive aesthetic and qualitative judgements of finished objects. High-quality objects are typically regarded as skilfully made, and the complexities underlying this association are rarely discussed (Kuijpers 2015). Nowadays, archaeometallurgy is an established field of research increasingly integrated and engaged with central issues in archaeology (Roberts and Thornton 2014). Notwithstanding, accepted interpretations of the social personae of specialist metalworkers and their knowledge and skills remain largely unchallenged by archaeometallurgical data (Kienlin 2013; Killick and Fenn 2012). Consumption and materiality studies are rampant, while production and the properties of material that objects are made of play a remarkable marginalised role (Ingold 2007; Martinón-Torres and Killick 2015).

The research undertaken by Kienlin (2007, 2008a, 2008b, 2010; Kienlin *et al.* 2006) is one of the few attempts to explore prehistoric metalworking from a theoretically informed approach coupled with an in-depth and comprehensive sampling of prehistoric artefacts. In this manner, he challenges the gap between the social archaeologists’ interpretations of metalworking and the material scientists’ body of factual data (Pollard and Bray 2007; Thornton 2009). Not only is this a considerable step forward in our understanding of prehistoric metalworking, it also makes possible a subsequent study of skill.

Kienlin’s work together with Junk’s (2003) investigation of early Bronze Age torques convincingly show that prehistoric metalworkers were knowledgeable about the workability of varying metal compositions; though it remains unclear to what extent. At the same time, their findings posit the question how this knowledge, needed to perform knowledgeable practice (*i*.*e*. skill), came into being in the first place. A more analytical and structured exploration of metalworking skill is preferred. Such an approach would define more precisely what this skill entailed, what it was based on, how it was applied, and to which extent skills can be read from the prehistoric objects.

## Skill and a Sensory Categorisation

Compositional analyses and the intentionality of certain copper-compositions are a constant topic of debate (*e*.*g*. Butler and van der Waals 1964; Bray *et al.* 2015; Earl and Adriaens 2000; Kienlin 2008b; Lechtman 1996; McKerrell and Tylecote 1972; Mödlinger and Sabatini 2016; Mödlinger *et al.* 2017a; Mordant *et al.* 1998; Northover 1989; Ottaway 1994; Pare 2000). I turned to contemporary metalworkers to inform whether the discussed differences actually mattered in terms of handling the material.^1^ Asking about the manner in which alloying decisions are made, I noticed that for most bronze objects it is not necessary to be precise about the alloy (*cf*. Northover 1989, p. 114). Rather, the metalworker aims at certain qualities like hardness, workability or even sound (see below). Quantities tend not to be measured carefully but, quoting Holger Lönze, ‘I chuck a good extra lump of tin into the mix if I am after hardness. It is guesswork at that stage [alloying] and you cannot tell the tin content from the molten metal. It is obvious from the colour afterwards.’ It seems that alloying takes place on the basis of *approximation* and is aimed at a compositional *range* in which the alloy behaves in a certain manner. An observation that allows us to rethink how to interpret compositional analyses.

The detailed metallurgical studies of the effects of certain elements on copper are possible *because* we recognise them as separate elements. Consequently, scientists are able to think of these elements as causational and examine the effects independently, and under laboratory conditions. The heuristic value of the scientific method is dependent on a modern, atomistic, insight of metals. These descriptions seem to have little significance for a craftsperson however, and in line prehistoric metallurgy. Many of the affected properties of copper that metallurgists have laboriously recognised may not be directly relevant to our understanding of prehistoric metalworking since the perceivable effect is too small, or too unpredictable to be observed by prehistoric metalworkers, let alone associated with a specific type of ore or metal composition (Coghlan 1975, p. 79; Kienlin 2008b, p. 252; Kuijpers 2013, pp. 142–43). This leads to a paradoxical conflict that increasing preciseness and accuracy, while a worthwhile scientific endeavour of itself, can potentially obstruct exploring the material from a craft perspective. By no means am I arguing that scientific analyses are incapable of shedding light on questions about prehistoric craft and skill. But one need to look at them where they quite literally, make sense.

Let me explain this by means of the example of the Central European Early Bronze Age. Apart from silver, gold and tin, there is at this time no unequivocal evidence that any of the commonly measured elements in copper were known to a prehistoric metalworker as a *separate* metallic element, and only tin was widely used for alloying. In the case of arsenic, because of the effect on the mechanical qualities and change in colour of copper, and the distinct white smoke and garlic odour when smelted (see below), there is a high probability that the different behaviour of arsenical-rich copper was recognised and utilised (Kienlin 2010, p. 18 with references; Lechtman and Klein 1999; Pearce 2007). However, nickel, antimony and silver share some of these effects in terms of colouring and hardening copper (Cheng and Schwitter 1957; Junk 2003, p. 27), all of which are found in the same ores (see below). For antimony, it has even been argued that it behaves *like* arsenic (Biringuccio 1990, p. 106; Scott 2011, p. 96). On top of this, arsenic-rich ores (tennantite) and antimony-rich ores (thetrahedrite) are hardly distinguishable from each other. It is therefore unlikely that these ores were recognised as separate materials, let alone that arsenic would have been recognised as a separate element, like tin, from other elements.

A reasonable assumption therefore is that arsenic, antimony, nickel and silver were all understood as one and the same ‘thing’ corrupting the normal qualities of pure copper, in a variety of ways. A sixteenth century example of this commingled understanding can be found in Agricola. In this period, there is considerable confusion around the group of arsenides. They appear to be lumped together under the term cadmia and because Agricola describes the garlic odour and corrosive qualities of cadmia (Agricola 1950, p. 113; Agricola 1955, p. 8), there is no doubt that arsenic is involved. Later, it was found that cadmia were forms of zinc, cobalt and arsenic. There is even the possibility that arsenic and tin may have been understood as ‘similar’, which can be inferred from another sixteenth century source on metallurgy where it is noticed that ‘orpiment and arsenic act in almost the same way as do tin and mercury’ (Biringuccio 1990, p. 105).

To a craftsperson, it is not a necessity to precisely understand what causes different raw materials to perform in a certain manner and why. What matters is that they recognise these differences and act upon it. This is a small but important nuance. It allows the archaeologists to look for skilled behaviour without presupposing technological knowledge.

Thus, instead of assuming that prehistoric metalworkers knew metallic elements and compositions, it is better stated that they were responsive to the recognisable behaviour of certain copper-compositions. Rather than seeing an opposition between the above two types of understanding (objective and explicit versus subjective and embodied), which inevitably seems to result in scholars that entrench on either side of this dichotomy (*e*.*g*. Dobres 2006; Ingold 2000), I take them as nothing more than a different choice of metaphors to describe similar material processes and properties.

After all, the qualities and behaviour of a material are a sensorial reading of the properties from which they stem. These two knowledge identities must be compatible with each other; they only make use of a different type of categorisation. Science and craftspeople are not describing different realities; they are simply describing reality differently. What is needed in our analysis of skill is a method through which to access this qualitative dimension using the quantitative data and measurements tools available. In the remainder of this paper, this idea is implemented through the analytical approach of perceptive categories.

## Method Description

### Perceptive Categories: a Sensory Update to the *Chaîne Opératoire*

Advances in understanding prehistoric metalworking skills are most likely made by adopting the *chaîne opératoire* approach (Vandkilde 2010, p. 905). As argued, this method is in need of a sensory update to incorporate skill and for this I make use of perceptive categories. A perceptive category transcribes those aspects of the material that are recognisable and relevant to craftspeople, to detailed scientific measurements; allowing for empirical validation of these perceptive categories in a dataset.^2^

By definition, this means that the perceptive category itself is interpretive as not all sensate features of a material are equally interesting. Presupposing that the constructed categories could have been noticed by prehistoric metalworkers (hence *perceptive* categories), I use them to organise and analyse the data. The distinctions made, however, are unmistakably etic constructs.

Experiments, contemporary craft and historical sources all hold valuable information on metalworking skills and this knowledge is used in the construction of perceptive categories. What I have attempted to draw from these sources is not an analogy but an understanding of ‘metalleity’ (Huxham 1753, p. 859). A useful word re-introduced by Bray (2012) to emphasise that metal constitutes a package of attributes which are available for human society, stressing the manner in which metal *behaves* and how this is perceptible—and thus understood—by craftspeople (Untracht 1969, pp. 5–6). As such, it is a useful concept to balance the atomistic insight of metal in contemporary sciences with the distinct appreciation craftspeople have of their material.

**Fig. 1:**
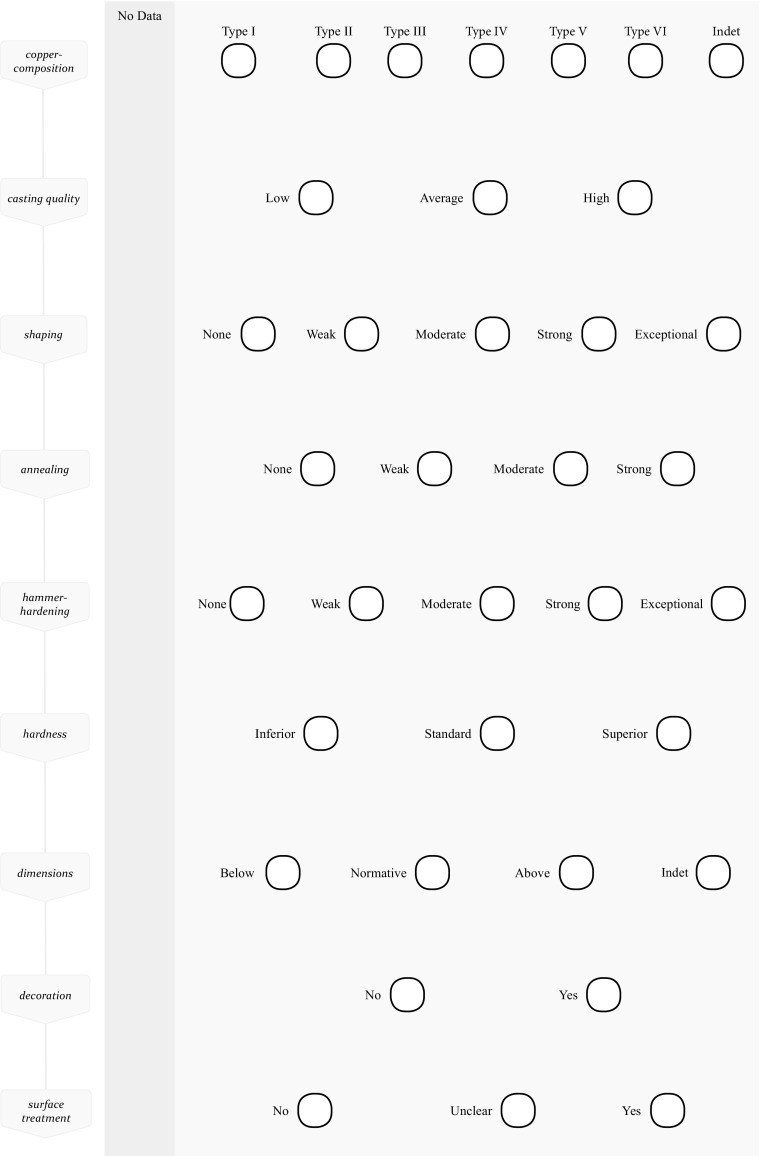
The *chaîne opératoire* typically only lists *what* choices were made during production (the vertical steps on the left). The recognition of different types of metal and *how* techniques were applied are added in the form of perceptive categories (horizontal nodes). This makes it possible to analyse skill

The perceptive categories subsequently are incorporated in a traditional *chaîne opératoire* to systematically analyse the production processes and make comparisons. They are the nodes on the horizontal axis, representing the possible relevant categories and applications of a material or technique (Fig. 1). This effectively updates the *chaîne opératoire* into a network of relational data that theoretically holds 151.200 possible paths to follow, starting with the type of metal and ending at surface treatment. Through these, it is possible to move beyond technology and to map the recognition of material, *how* techniques were applied, and whether this was done in response to the material or earlier steps in the *chaîne.* This is where skill becomes visible.

Metalworking is narrowly defined here as the process in which raw metal is transformed to an object. Mining, smelting, use and deposition are all part of the life cycle of metal, but not included in the metalworking *chaîne* that I present here. The metallographic and compositional data that underlies the construction of these *chaîne opératoires* was gathered by Tobias Kienlin (Kienlin 2008a, 2008b, 2010), while the translation into perceptive categories is the key element of my own work (Kuijpers 2018). Space does not allow me to work through all the perceptive categories that are used in Fig. 1 and I will limit myself to clarifying how the first six on composition have been constructed*.* Casting quality, the amount of deformation (either through shaping or the final hammer-hardening), and annealing are given in Tables 1, 2 and 3.

**Table 1:** Perceptive categories of casting-quality based on the amount of porosity and oxides in metallographic samples. A description of the metallographic structure and the manner in which this is perceptible to a craftsperson are given in the first two columns. On the right the amount of porosity (M1) and oxides (M2) as found in the metallographic samples of axes used for this research and published in Kienlin (2010, Fig. 4.10, 7.9, 7.10; 0 = absent, x = medium amount, xx = high amount)

Casting-quality				
Metallographic structure	Perceptible qualities	Perceptive category	Amount of porosity (M1)	Amount of oxides (M2)
Pores and oxides throughout the sample and in large quantities. Pores are often over 50 μm and up to 100 μm	High porosity may be visible on the surface in the form of pores or can be surmised from the low sonorous qualities of the metal when tapped/hammered. Metal is difficult to work due to oxides. Cracking and fracturing are likely	Low-quality cast	xx (xx) x xx	xx (xx) xx x
Some pores and oxides are present in the sample. Pores are mostly medium sized 30–50 μm	Some porosity and oxides are present but unlikely visible. The metal can be worked with no additional problems and the metalworker likely has little notion of what is going on in terms of porosity and oxides	Moderate-quality cast	x x (x) (x)	x (x) x (x)
Porosity and oxides are rare in the sample. Pores are small (< 30 μm)	Low amount of porosity and oxides. This may be noticeable to a metalworker through the good workability of the axe (malleable) and the good sonorous quality. The metal ‘rings’ when hit. Little risk of cracking or fracturing due to porosity and/or oxides	High-quality cast	0 0 x	0 x 0

**Table 2:** Perceptive categories of hammering based on the amount of reduction in the metallographic sample. The first column describes the changes in microstructure that can be read from metallographic samples. The following three columns transcribe this information to perceptible changes to the metal, the interpretation of these changes and the subsequent perceptive category that is used to organise the data. The last column on the right gives the amount of reduction in percent as found in the metallographic samples of axes used for this research and published in Kienlin (2010, 2008b). The amount of reduction is the reduction in thickness of metal during a single round of hammering, without annealing

Amount of hammering (shaping and hardening)				
Metallographic structure	Perceptive changes to the metal	Interpreted as	Perceptive category	Amount of reduction in %
As-cast structure visible (*e*.*g*. dendrites). Few slip lines on the surface	Removal of feeders, casting seams, flashing and other irregularities	Very light hammering. Not a shaping or hardening operation. Cleaning up as-cast axe	None	0 5 10
Slip traces, duplex slip, (several systems of) strain lines	Changing shape of axe’s body and blade. Achieved with little effort. Metal gives little resistance	Mostly a shaping operation. Little risk of cracking the metal	Weak	15 20 25
Deformation of grains	Metal noticeably harder to deform. Resistance is felt as the metal hardens. Sound changes	Shaping and hardening operation. Some risk of cracking depending on the type of metal.	Moderate	30 35 40
Heavy deformation of grains. Grains elongated. Pores deformed	With considerable hammering, no significant deformation. Resistance is clearly felt and hammer ‘bounces’ of the metal. Sound of hammer hitting the metal goes up (higher tone)	Mostly a hardening operation as shape becomes ‘set’. Clear interest in hardness of the axe. Ample risk of cracking and some chipping of the cutting edge	Strong	45 50
Extreme elongation or blocky structure. No grains discernible anymore. Pores heavily deformed or closed	Forceful hammering leads to little visual change in the shape of the axe. Hammer bounces strongly and creates a high pitches sound	Pushing boundaries. Hardening to the point where for most types of metal cracks are likely to appear. Also the risk of chipping or fracturing	Exceptional	> 55

**Table 3:** Perceptive categories of annealing intensity based on recrystallization and homogenisation in the metallographic sample. A description of the metallographic structure and the manner in which this is perceptible to a craftsperson are given in the first two columns. The three columns on the right show the amount of recrystallization (M8), homogenisation (M11) and annealing intensity (M12) as found in the metallographic samples of axes used for this research and published in Kienlin (2010, Fig. 4.10, 7.9, 7.10; 0 = absent, x = medium amount, xx = high amount)

Annealing-intensity					
Metallographic structure	Perceptible	Perceptive category	Recrystallization (M8)	Homogenisation (M11)	Intensity (M12)
Dendrites or casting grains, no recrystallization	–	None	0	–	–
Partly recrystallized or local recrystallization. No homogenisation	Low temperatures (< 500 °C). The metal does not glow, or glows weakly orange-coloured	Weak	x xx	0 0	(x) 0
Full recrystallization, partly homogenised	Medium temperatures (~ 500–600 °C). Metal glows red	Moderate	xx	x (x) (x) x	x (x) x (x)
Full recrystallization, full homogenisation. In some cases no coring left	High temperatures (600–700 °C and up). Metal glows cherry red to orange. Risks hot-short	Strong	xx	x xx (xx) xx	xx x (xx) (xx)

### Perceptive Categories for Copper-Compositions

The first step in this *chaîne opératoire* is the raw material. I deliberately avoid the word ‘choice’ here. What I intend to analyse is whether different copper-compositions were recognised, from which we should not uncritically infer that they were deliberately chosen or alloyed. I assume that most metalworkers recycled scrap material or worked with impure ingots, and that re-melting took place at the expense of control over composition (Bray *et al*. 2015; Bray and Pollard 2012). To adequately recognise the qualities and behaviour of the varying coppers *at hand* would thus have been an important skill as it largely determines how the material can be worked, or how it should be alloyed (*cf*. Hiorns 1912, 215).

Colour plays an important role here because it provides the metalworker with a perceivable quality of the material that allows for differentiating between copper-compositions (Hansen 2013; Kienlin *et al.* 2006; Mödlinger *et al.* 2017a; Pearce 2007). Historical sources leave little doubt that colour was a key indicator of specific metals and their purity (Agricola 1950; Guettier 1872). This, therefore, relates to an (pre-scientific) understanding of compositional differences.^3^ This particular quality of metals has long been recognised as relevant, and is often invoked to interpret metalworking (Leusch *et al*. 2015; Hosler 1995; Jones 2004; Jones and MacGregor 2002; Radivojević *et al*. 2013; Smith 1975; Villegas and Martinón-Torres 2012). However, there have been few attempts to quantify the relation between composition and colour, and typify metal on the basis of these findings (Berger 2012; Chase 1994; Devogelaere 2017; Fang and McDonnell 2011; Mödlinger *et al*. 2017b). This lack of clearly identified types of metal *as relevant for a metalworker* prevents a systematic analysis of skill from happening. Consequently, I have chosen colour as the main referent of the perceptive categories for copper-composition, but other characteristics such as increased hardness or extreme brittleness are also taken into account.

The copper-tin compositions that are thought relevant from a craft perspective are 0–5% (red), 5–12% (yellow), 12–20% (golden) and 20% > (silver). With regards to copper-compositions that contain one or more of the elements antimony, arsenic, nickel and/or silver, which I lump together for reasons discussed above, the following groupings are surmised: 0–3% (red), 3–7% (orange) and 7% > (white). All of these may additionally contain traces of other elements, but these are considered irrelevant. The discussed colours are based on polished samples. I make no claims that any of these compositions were intentionally alloyed, some certainly were, some were not. For some, we might never know. Co-smelting and arsenic loss complicates the discussion for arsenical bronzes (Mödlinger *et al*. 2017a; Mödlinger and Sabatini 2016). But even when it is certain that the alloy was intentional, as is the case with tin bronze, the amount in which the element is present may not have been a deliberate choice. Low-tin bronzes can also be the result of unintentional loss of tin due to frequent re-melting (Wang and Ottaway 2004, p. 77).

**Fig. 2:**
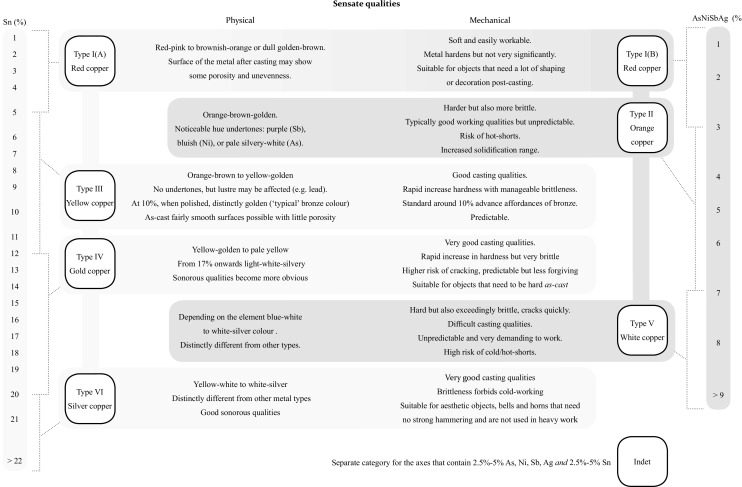
Six perceptive categories of copper-compositions based on their perceptible qualities that are relevant for a metalworker

What I am advocating is that these particular copper-compositions have such specific metalleity that they are (easily) distinguishable from each other through sensory cues only. They, therefore, potentially were recognised as unalike materials, each which its own distinct behaviour and ‘rules’ of how they may be worked (*cf*. Junk 2003, p. 4). They underlie the six perceptive categories for the ‘raw’ material that the prehistoric metalworker needed to work with, each of which is substantiated in more detail below (Fig. 2).

#### Metal Type I: Red Coppers

**Fig. 3:**
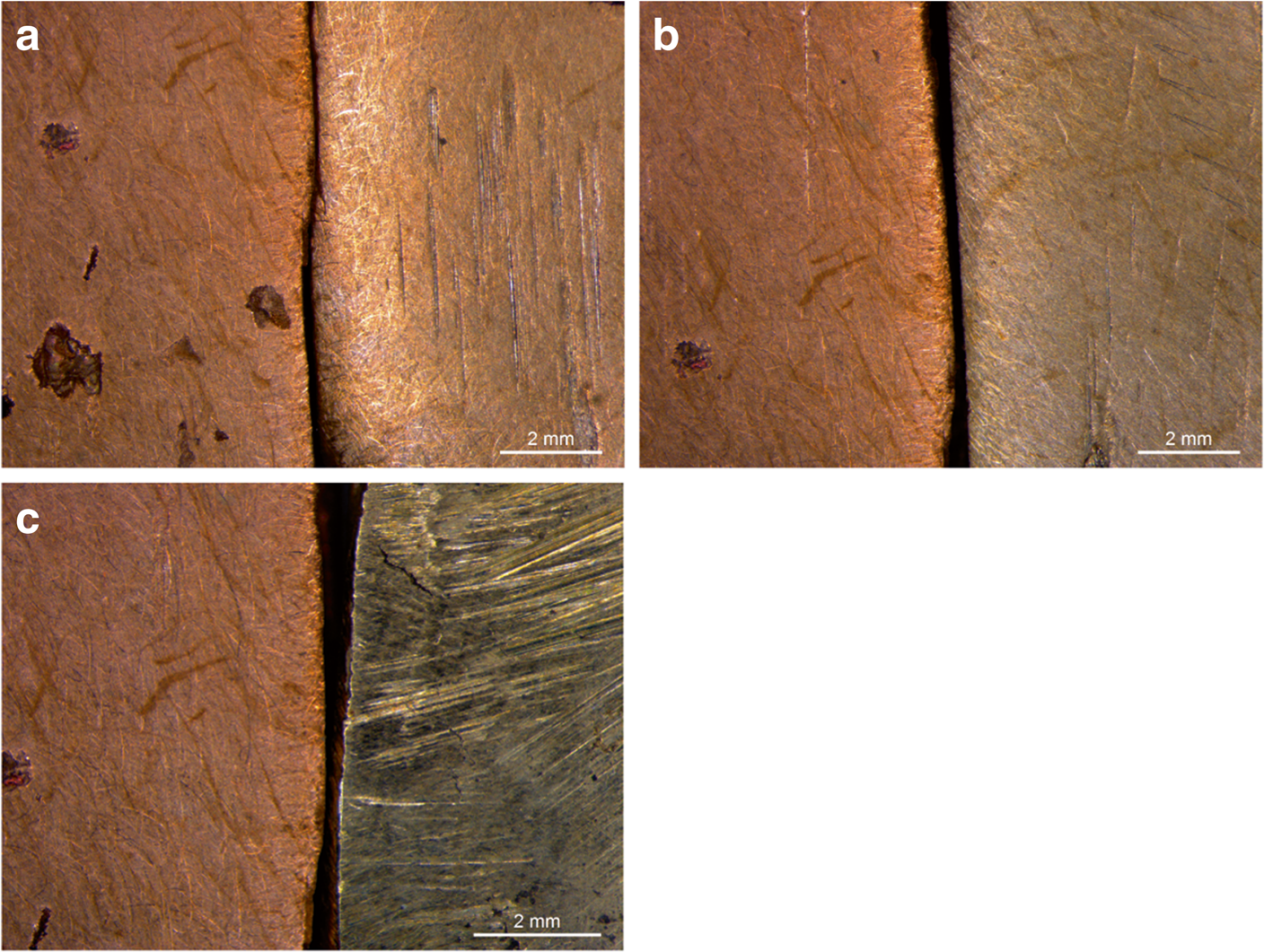
A range of samples to exemplify the colour differences due to the presence of tin, nickel and/or antimony, categorised according to perceptive categories. All are compared to a type I red copper (98.4 wt.% Cu) in the left of the picture. To the right in the picture are in **a**) type II orange copper (Cu 95 wt.%, Sb 4.4 wt.%); **b**) type V white copper (90.1 wt.% Cu, 5.2 wt. % Ni, 3.7 wt.% Sb); **c**) type III yellow copper (Cu 90 wt.%, Sn 10 wt.%). Samples are roughly polished to counter reflection which complicates capturing the colour on camera (Photographs by the author)

Any copper-composition with less than in total 3% arsenic, antimony, nickel and/or silver, or less than 5% tin, is considered to be type I metal.^4^ From the perspective of a metalworker, type I metal behaves like copper. The colour variation within this group is red to red-orange-brown but all close to the red of pure copper (Mödlinger *et al*. 2017b) (Fig. 3). The category entails both pure and ‘dirty’ copper (Lechtman 1996), as well as what some scholars would consider tin-bronze (Pare 2000, p. 2). The small variances within these type I copper-compositions would only have been perceivable to an extraordinarily attentive metalworker, if at all.

The differences in hardness of compositions with a combined weight of < 3% (As/Sb/Ni/Ag) are likely not noticed (Coghlan 1975, p. 79; Kienlin 2010, p. 78). In the case of tin, everything below 5% tin is a ‘soft’ copper that can be worked and/or shaped considerably, either by hammering or decoration techniques like repoussé (Lönze pers. comm.). Despite the relative softness of this material, it would still need regular annealing. Experiments have shown that low tin-bronzes (2% Sn) with little porosity can be reduced up to 80% without cracking (Nerantzis 2012, p. 240). Wang and Ottaway (2004) report micro-cracks on the surface of a 2% tin-bronze at 60% reduction.

With these fairly ‘pure’ coppers (especially 97–100% Cu)^5^, it is hard to produce flawless castings due to high gas uptake and mediocre fluidity of the material (Coghlan 1975, p. 65; Scott 2011, p. 82). A cast from this material is typically high in porosity and oxide inclusions, which may impede further working since the amount of hammering a copper can withstand is partly dependent on the porosity of the cast. Hence, despite its malleability, type I metal is considered to be a demanding material to work with because of its sub-par casting quality.

#### Metal Type II: Orange Coppers

Type II are the compositions that hold in total 3–7% arsenic, antimony, nickel and/or silver. This category is partly based on Junk’s findings that prehistoric metalworkers recognised and separated ‘ingot torque copper’, typically holding around 4–5% arsenic and antimony, from ‘low-impurity copper’ (Junk 2003, pp. 16, 169–174).

Junk undertook laboratory experiments with a composition of 96% copper, 2% arsenic and 2% antimony and found the hardness to be almost similar to that of pure copper in the as-cast stage (50 HV [Hardness Vickers]), but rapidly increasing if cold-worked (Junk 2003, p. 172). Hardness values of this metal at 50% reduction differ enough, compared to pure copper at the same reduction rate, to be detectible (Kuijpers 2018). Additionally, the solidification range of ternary Cu-As-Ni compositions increases and it is therefore assumed that type II coppers generally have fair casting qualities (Lechtman 1996, p. 85). The exact qualities of this metal type are very dependent on the amount of each specific element, however (see below). And the combination of arsenic and antimony affect copper more strongly than either of these elements alone (Archbutt and Prytherch 1937; Junk 2003, p. 32).

The colour of type II metal differs from the red of pure copper, but we cannot speak of a distinct colour change (Fig. 3a). Depending on the weight of specific elements, certain undertones are visible. Nickel and arsenic will give a greyish colour and ‘cool’ hue to copper, while antimony provides a salmon-red and ‘warm’ feeling to the metal (Guettier 1872, p. 109; Mödlinger *et al*. 2017b). Largely, all of the compositions in this group share shades of orange. As a result, the metalworker would have had few perceptible clues about the different behaviour of this copper prior to handling it. The behaviour of type II material might thus appear ‘random’ and, subsequently, difficult to appreciate from a craft perspective. Hence, despite the likelihood that the copper is positively affected by the presence of certain elements, this group of copper-compositions is best defined as an unpredictable, and accordingly risky material to work with.

#### Metal Type III: Yellow Coppers

Type III are all compositions that contain 5–12% tin. This is generally known as bronze in its traditional sense. The colour runs from orange-yellow to the typical yellow-golden colour (from around 9% tin) and is easily distinguishable from the types above (Berger 2012; Fang and McDonnell 2011; Mödlinger *et al*. 2017b) **(**Fig. 3c).^6^ There are no undertones visible, although elements like lead will affect the strength of the lustre making the metal appear dull (Devogelaere 2017; Guettier 1872, p. 85).

This type of metal is recognised for its good casting qualities because of the fluidity and relative slight gas uptake when molten (Coghlan 1975, p. 67). Resulting casts therefore typically contain little porosity.

Type III metal is malleable and can be cold-worked without problems. An as-cast 10% tin-bronze is almost double the hardness of pure copper (Lechtman 1996, p. 488) and will harden considerably upon hammering, without becoming brittle too quickly (Berger 2012, p. 26; Wang and Ottaway 2004). With frequent annealing, this metal can withstand a great amount of reduction unless other elements, like lead, are present in too large quantities (Nerantzis 2012, 2015). Moreover, during hammering, the sound and feel of a hardened tin-bronze is an easy to recognise cue attesting to the metalworker that certain hardness is reached and annealing necessary to prevent cracking (Untracht 1969, p. 246).

Type III copper-compositions can be summarised as a metal with overall good qualities, affording an expedient combination between fluidity, workability, hardness, strength and perhaps most importantly, predictability.

#### Metal Type IV: Gold Coppers

Copper-compositions with 12–20% tin are categorised as type IV material. The behaviour of this type of metal differs from type III mostly on mechanical aspects and less so on physical characteristics. In colour, they are close to the previous group and the golden yellowness of bronze is at a maximum between 11 and 13% tin (Mödlinger *et al*. 2017b). From ≈ 16% upwards, the metal will become noticeably lighter and paler towards a grey-silver tint.

Due to its excellent fluidity, this material behaves well when poured, typically producing high-quality casts with little porosity. On top of this, it will be a hard as-cast metal and this quality can be stressed even further by hammering (Nerantzis 2015, p. 333). However, this easily leads to fractures as the metal quickly embrittles, even with frequent annealing. Berger (2012, p. 26) found 11 and 12.7% tin-bronze to show cracking with moderate reduction. Wang and Ottaway (2004, p. 71) argue that bronzes with 15% tin cannot be reduced in thickness by cold-working beyond 30%. Nerantzis, however, writes of 15% copper-tin that he hammered up to around 40% reduction. Only in the second round of hammering, where deformation went above 50%, did cracks appeared across the thickness of the samples (Nerantzis 2012, p. 243). Despite these differences, the general bent of this metal is that it is noticeably harder, but also brittle. This makes type IV copper-compositions a less forgiving material than the comparable type III and, therefore, more demanding to work with.

#### Metal Type V: White Coppers

There is strong evidence suggesting that copper with high amounts of corrupting elements was recognised. This is best exemplified by a small group of axes identified by Tobias Kienlin that are incorporated in this research (Kienlin 2008b; Kienlin *et al*. 2006). Type V metal is partly based on this finding but more inclusive. Containing a total element weight ≥ 7% of arsenic, antimony, nickel and/or silver, type V material represents a special metal. Little is known about this material’s behaviour. Research is typically focussed on the effect of independent elements rather than the conglomerate in which they appear in prehistoric metalwork. Nonetheless, it is apparent that this material is easily distinguishable from the rest. A high amount of either arsenic or nickel (or both) causes a distinct silver-white colour (Fig. 3b**)** (Berger 2012; Lechtman 1996; Mödlinger and Sabatini 2016; Mödlinger *et al*. 2017b). Probably for this reason in the recent past alloys of arsenic and copper were known under the name of ‘white coppers’ (Biringuccio 1990, p. 54; Cheng and Schwitter 1957, p. 361; Guettier 1872, pp. 117–19). This colour is so distinct that it is often still visible even when the metal is patinated (the patina being grey-blue instead of the typical green hues). Besides colour, type V metal has other easily recognisable qualities.

Arsenic can significantly improve work-hardening properties (Lechtman 1996, p. 492; Nienhuis 2009, p. 19) though too much of it makes copper exceedingly brittle and porous (Charles 1967, p. 21: Ottaway 1994, p. 130). What exactly is too much is a matter of debate: 4–5% (Northover 1989, p. 117), 6–7% (Kienlin 2008b, p. 255) or over 7–8% (Cheng and Schwitter 1957, p. 361; Junk 2003, p. 22; Lechtman 1996, p. 481). Antimony is another cause of brittleness and makes copper liable to hot-shorts (Hiorns 1912, p. 21; Junk 2003, p. 28; Merkl 2010, p. 21; Scott 2011, p. 96).^7^ The workability of white coppers is therefore poor, and when hammered they will fail easily.

As with type II metal, the exact behaviour of the material is difficult to predict. For example, high nickel may give the appearance of a white copper but not ‘act’ as such, while high antimony causes extreme porosity. Problems with hot- and cold-shortness plagues this metal (Hiorns 1912, p. 21; Junk 2003, p. 28; Makar and Riley 1985, p. 6; Merkl 2010, p. 21). Consequently, although type V material is easy to recognise, it must have been a particularly demanding material to work with (*cf.* Kienlin 2010, p. 155).

#### Metal Type VI: Silver Coppers

Categorised as type VI material are the copper-compositions containing ≥ 20% tin. Their colour is yellow-white to white-silver (Mödlinger *et al*. 2017b). This material is extremely brittle and unsuited to hammer-hardening but carries completely different qualities from the other types discussed, such as their sound (Hiorns 1912, p. 215: Scott 2011, p. 134–35). Given this sonorous quality, these alloys are typically used for bells or horns (Northover 1989, p. 115), and in modern day metallurgy it is accordingly known as bell metal (McCreight 2010, p. 10). This type of metal is unusual in the prehistory of Western Europe, and absent in the dataset I worked with.

## Discussion

### Metalworking Skills

**Fig. 4:**
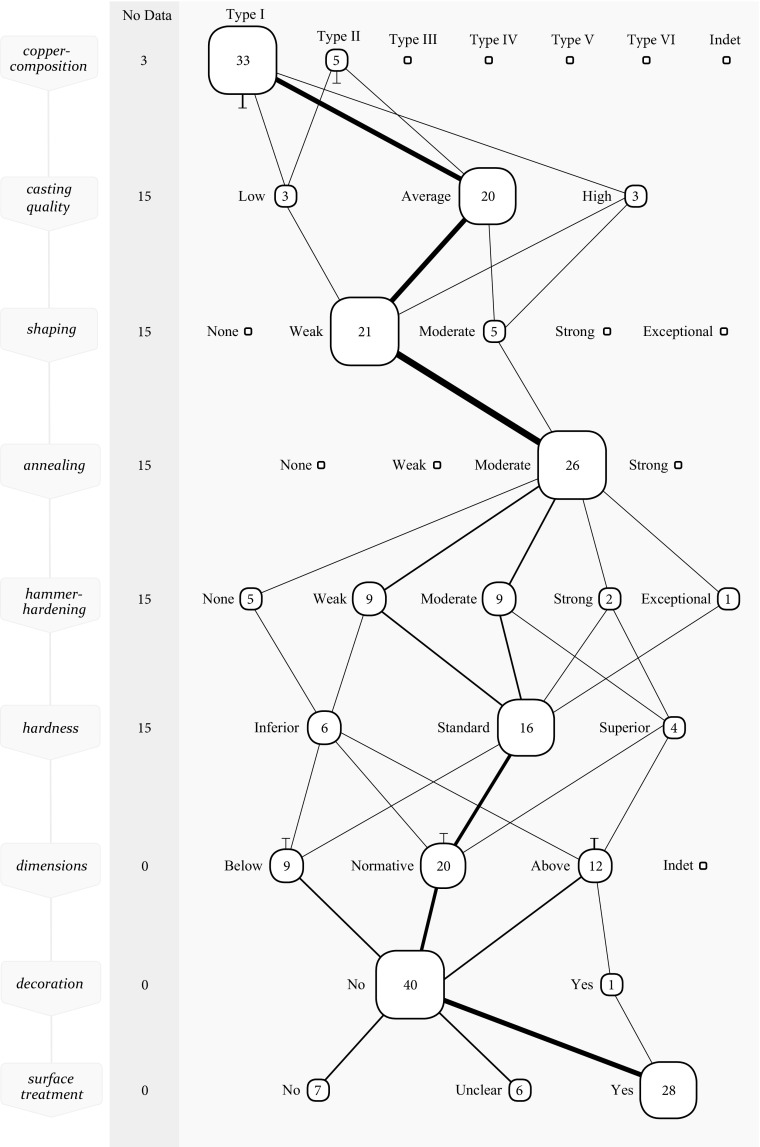
Collective *chaîne opératoire* for the LCA axes. The nodes represent the different possibilities (perceptive categories). While it is not possible to follow an individual axe in this *chaîne opératoire*, it grants an insight in the most commonly applied steps in the production of a prehistoric axe. The number displayed within each node represents the absolute number of axes that have been categorised for that specific perceptive category. The node-size is based on the percentage of axes that are in the node in relation to all axes in the neighbouring nodes. This makes it easy to notice how often a technique was applied in a certain manner compared to the other possibilities. When an axe proceeds to the next technological step, this is shown by a link between nodes. The line thickness of the link represents the percentage of axes that move from one node to a node in the next step in relation to all axes that proceeded to the next step. Lines that stop or begin between steps represent the removal or re-entry of axes for which no data for the next or previous step was available. If no data is available for a particular step, the axes are listed in the ‘no data’ column on the left

**Fig. 5:**
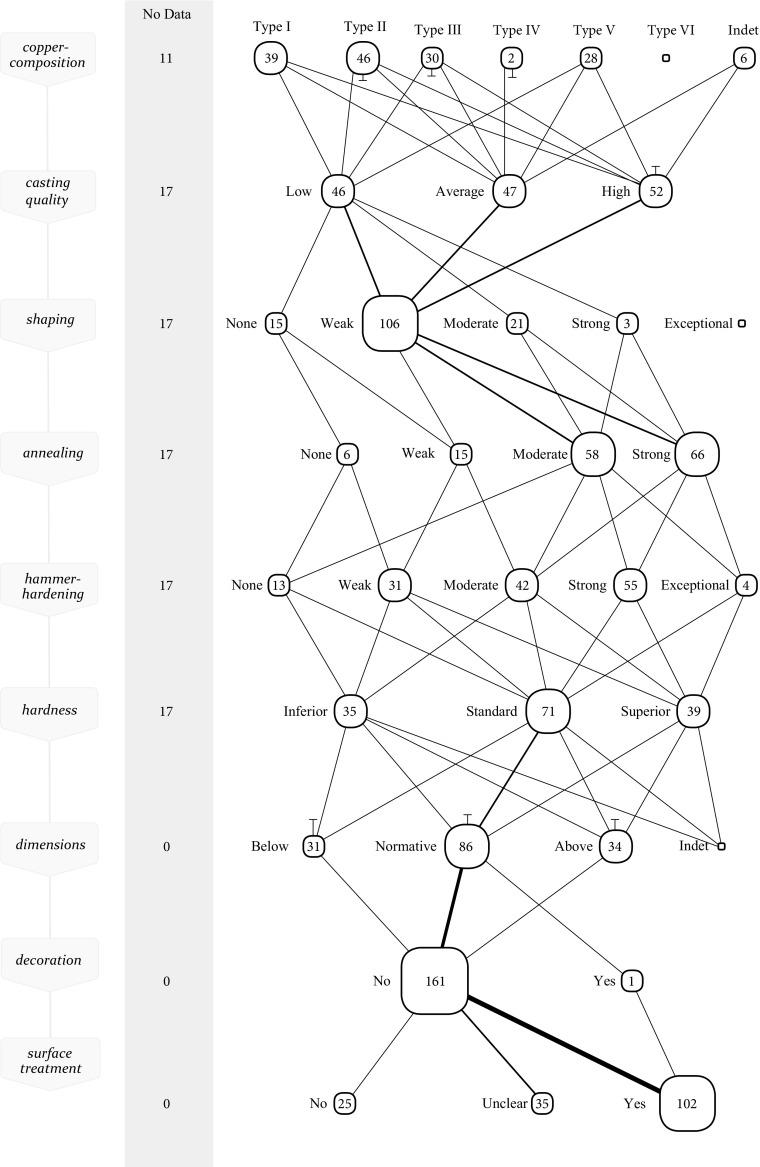
Collective *chaîne opératoire* for the EBA axes

In Figs. 4 and 5, the production processes are plotted of 41 Late Copper Age axes (hereafter LCA) (roughly dating to the late 4th millennium BC) and 162 Early Bronze Age axes (hereafter EBA) (2200–1900 BC) in a *chaîne opératoire* updated with the above proposed framework of perceptive categories. While this collective visualisation of all axes forbids to follow an individual axe, it grants an insight into the most common application of techniques, and the common links between techniques. This collective *chaîne opératoire* is thus particularly informative of the *general* procedure of manufacturing axes, because it shows the most travelled paths in practice from a plethora of options. This, then, is useful as it shows the norm to which individual axes can be compared. After all, a designation like ‘skilfully made’ inevitably is a comparison to a norm. It is important to make this explicit in order to substantiate skill in an empirical manner, and to avoid intuitive judgements (Dobres 2006).

Skill is not a fixed entity but lies in the recognition of and response to a material’s affordances, resulting in a wide array of possible outcomes. An inquiry into skill therefore asks for an investigation of each and every axe and the manner in which it was individually produced, and an understanding of the differences in quality that can be observed (Kuijpers 2017, 2018). Nonetheless, I will attempt to draw some general observations from the collective *chaîne opératoires* presented here, because it is possible to discern certain trends through time that are a result of the availability of the different types of metal and the ease at which they may be recognised by an attentive metalworker.

#### The Development of Skills Through Time

The majority of the studied axes from the LCA were made from red coppers (type I). Only a very small group of axes was made of orange coppers (type II) that would behave perceptibly distinct. From a craft perspective, metalworkers during the LCA thus encountered few difficulties as the metal generally behaved in a similar—predictably difficult—manner. This perceived similarity of the raw material may partly explain the straightforward working of this material, with little variation in how techniques were applied. The typical average casting quality of these axes is remarkable because type I coppers are susceptible to (heavy) gas uptake leading to high amounts of porosity and oxides. Principally, explanation of the average casting quality should be sought in a skilled application of melting and casting techniques, and the preparation of a well-made mould. Furthermore, the malleability of copper allows for some porosity to be ‘removed’ afterwards because strong hammering to some extent deforms oxides and closes porosity making them less visible in the metallographic sample (Kienlin 2010, p. 31). Despite a fairly standardised technology, some variation is visible, especially in *how* the technique of hammer-hardening was applied (Fig. 4). This is a result of varying levels of skill, but this will become more clear when compared to the working of this material in the succeeding period.

In the first half of the EBA, things became more complicated for the metalworker. Through the perceptive category approach, five distinct metal types are identified in this period. Whether this is the result of intentional trade and alloying, specific ore-deposits, or recycling does not matter for a study of skill. What I am interested in is to see how metalworkers responded to this variety of raw materials in this period. From the collective *chaîne opératoire*, one particular observation is evident: metalworking in this period was motley, to say the least (Fig. 5). Note, however, that there have been no changes to the technology itself. Production of the majority of axes in this period is similar to the LCA in terms of techniques used: casting, shaping, annealing, hammer-hardening (cold-working) and surface treatment. This standardisation is positively documented by Kienlin (2010, 2008b) and an increasingly remarkable observation given the vast social changes taking place in the 3rd millennium (Kristiansen *et al*. 2017), and in light of the fact that copper metallurgy of the late 5th and early 4th millennium did differ in applying hot-working (Kienlin 2008b).

*How* these techniques were applied varies greatly in the EBA, however. Does this mean that EBA metalworkers were able to recognise the different metal types, and how skilfully did they respond to the differences in material behaviours? For this, we need to look at some of the metal types independently.

#### Working Type I Metal: Red Coppers

The introduction and use of type II and type III metals in the first half of the EBA led to an overall higher standard in terms of hardness, compared to the LCA (Kienlin 2010, 2013). This is largely due to the fact that these materials afford greater hardness values (type II, and especially III). What is rarely discussed is how this introduction affected the perception of the traditional material, which is type I metal. In terms of hardness, the relatively ‘soft’ type I metal is at a disadvantage in this period, especially in the presence of type III metal. How did EBA metalworkers respond to this reality?

**Fig. 6:**
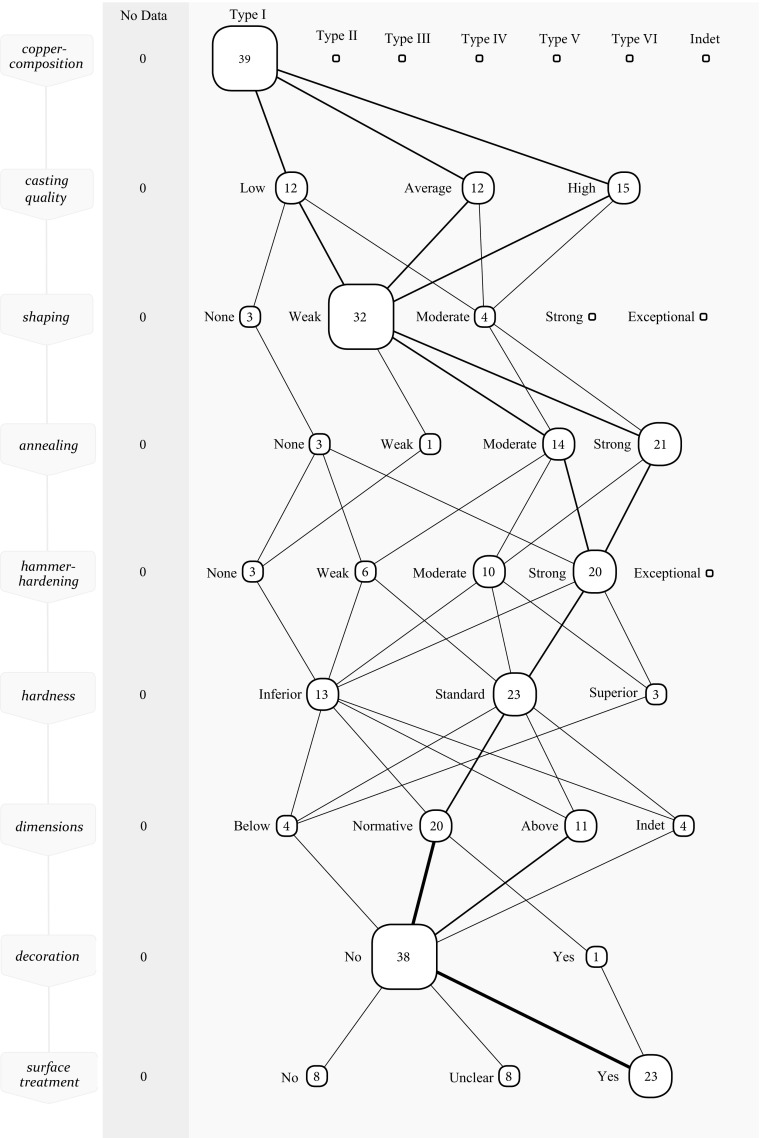
Collective *chaîne opératoire* for the EBA axes made of type I metal

One can observe that the hammer-hardening of type I metal has clearly shifted from weak/moderate in the LCA (Fig. 4) to moderate/strong in the EBA (Fig. 6). It appears that not only did metalworkers recognise the soft nature of type I material, they also responded accordingly by increasing the amount of hammer-hardening. Whereas a weak to moderate hammer-hardening sufficed to make ‘good quality’ axes in the LCA (*i*.*e*. between 93 and 134 HV), the same material is now in need of a more intense hammer-hardening in order to push the hardness towards the standard of the time of this period (*i*.*e*. between 138 and 199 HV) (see Kuijpers 2018 how this standard is calculated). Taking this evidence one step further, it seems that metalworkers were willing to take more risk. With stronger hammering, there is an increased probability that cracks appear. Even axes with low casting quality were regularly subjected to a strong hammer-hardening. Partly, this risk was tackled by means of an intense annealing to recover the workability. Here too a clear shift from moderate to strong can be noticed in the *chaîne opératoires.* Despite the strong hammer-hardening, there are four of these axes that were still of substandard hardness compared to the standard in the EBA. These are good examples of the craftsperson’s dependence on the qualities that a certain material affords.

#### Working Type III and IV Metals: Yellow and Gold Coppers

**Fig. 7:**
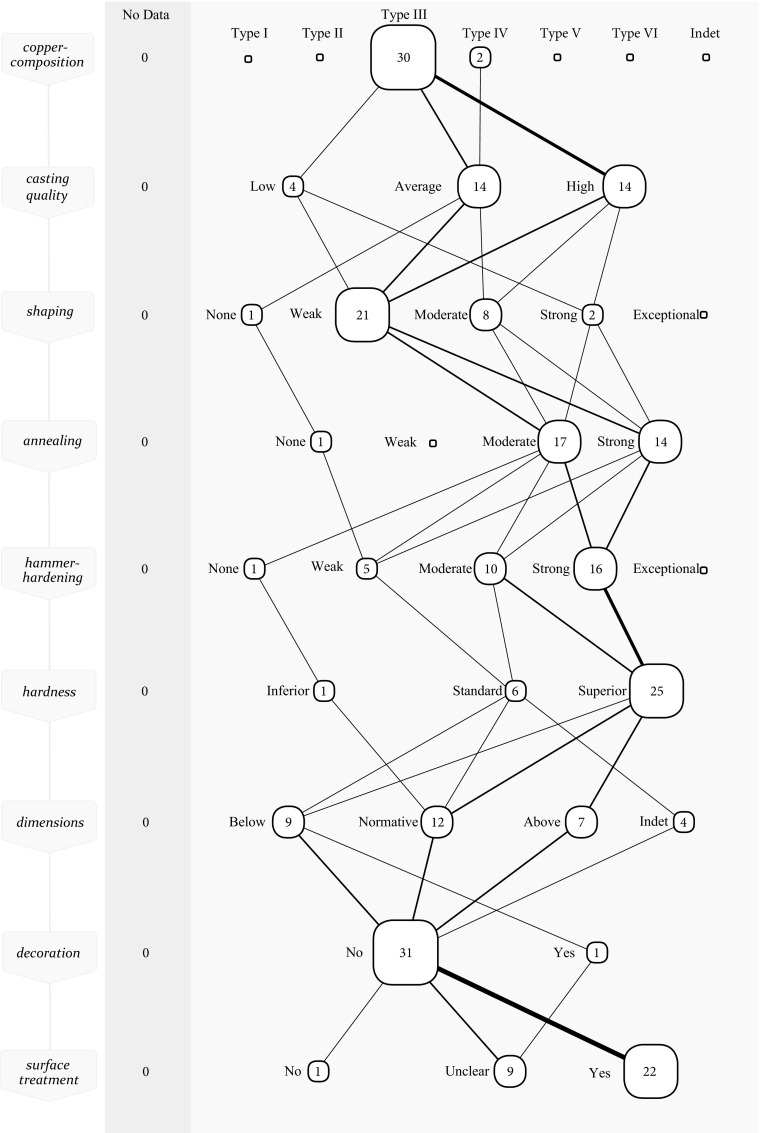
Collective *chaîne opératoire* for EBA axes made of type III and IV metal

Highlighting the axes made of type III and IV metal, there are a few noticeable differences in this collective *chaîne opératoire* compared to the other types of metal (Fig. 7). Metalworkers benefitted from the favourable casting qualities of this material and the ease with which they harden to high hardness values upon hammering. Apart from four exceptions, all axes are average to good quality casts. This also affected their subsequent workability as good castings are less prone to problems, and this was recognised. Several axes were shaped moderate to strong in one or more rounds of shaping and annealing. In the final hammer-hardening, there is little difference compared to the other types of metal. Yet, due to the inherent qualities of this material, a total of 25 axes are of a superior hardness in comparison to the standard of the time.

#### Type V Metal

**Fig. 8:**
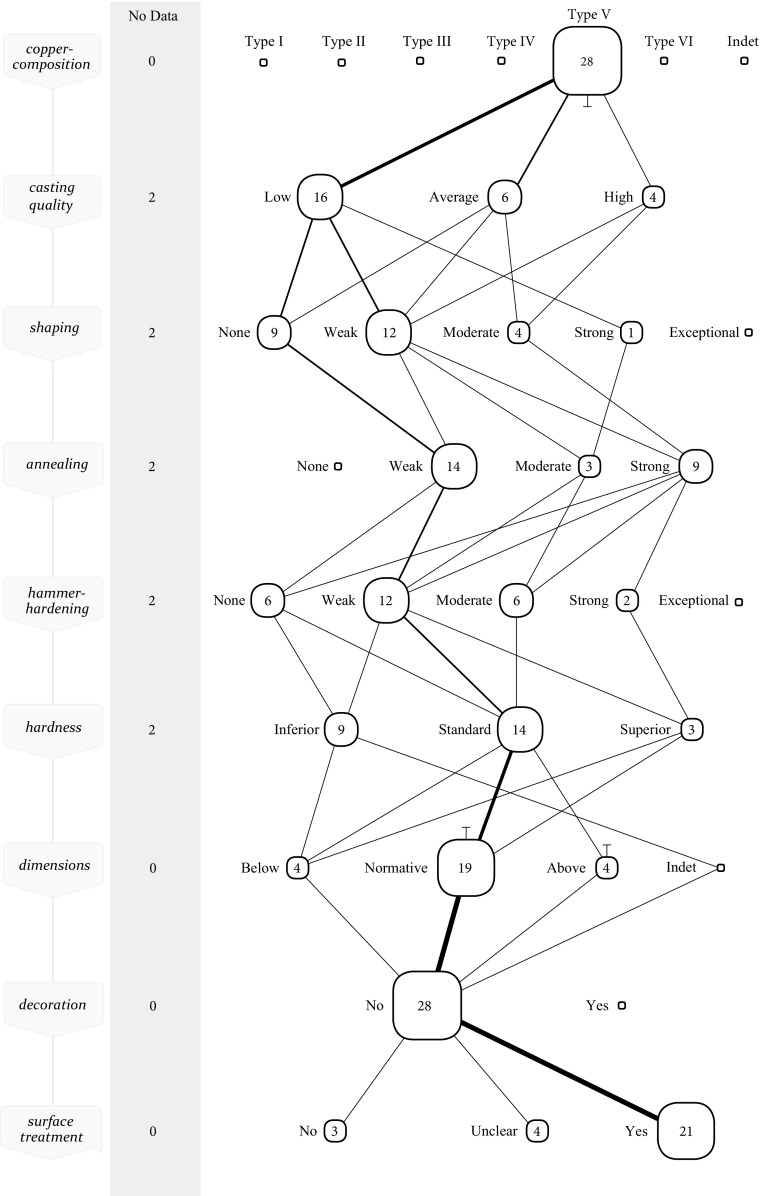
Collective *chaîne opératoire* for EBA axes made of type V metal

This material’s distinct behaviour and appearance would have made it fairly easy to be recognised by a metalworker and via the method of perceptive categories this can be demonstrated convincingly. There are 28 axes in the dataset with a copper-composition that is defined as type V metal. When combined in a collective *chaîne opératoire*, the axes made of this material show remarkable differences from other types of material (Fig. 8). The casting quality of these axes is typically poor, which is likely a result of the material rather than skill in the making of the mould (high amounts of antimony cause poor casts). Some metalworkers attempted to work this material in a standard manner,^8^ with varying success (see Kuijpers 2018 for the individual axes). A more common approach seems to have been to *avoid* hammering of this porous, unpredictable and brittle material. Out of 28 axes, 12 saw a weak hammer-hardening and 6 were not hammer-hardened at all. This is visualised in the collective *chaîne* where the most commonly travelled path has shifted to the left compared to type III metal. This confirms Kienlin’s observation of a group of axes from the Hindelwangen, Sennwald-Salez and Bohringen hoard (Kienlin 2006, 2008b) being worked in a distinct manner, but adds more axes, unrelated to these hoards, which were also worked differently from the norm.

Visualised in this updated *chaîne opératoire*, with the help of perceptive categories, the difference for the first time becomes exceptionally clear. But to speak of pattering would deny the diversity that is present. Whether a metalworker was able to recognise and respond to the material at hand is a matter of skill, and the diversity thus betrays varying levels of skill.

## Conclusions

Skilled crafting leads to a distinctive and intimate understanding of materials. Skill operates at the level where the qualities and behaviour of materials are understood through sensory engagement with them. To operationalise this craft perspective, I propose the use of perceptive categories.

A sensorial categorisation of material is distinct from the scientific one, but not separate from it. The difference is that craftspeople are focussed on the qualities of a material, while scientists are occupied with material properties. In line with this is the relevant level of detail. A craftsperson approximates, to the scientist it is accuracy and precision that matters. While this may come across as a critique of the scientific approach, it is actually good news for the usefulness of archaeometallurgical data. We now know that many of the older compositional measurements are imprecise, and that different techniques lead to varying results. This is problematic in a scientific sense, but not so much for the perceptive categories approach.

As an example, the perceptive categories for copper-compositions are given, and I explain why and how they would be recognisable and relevant to a metalworker. These categories likely need refinement and this is only a first attempt to work from a craft perspective. For instance, incorporating fuzzy logic would be a logical next step since it goes without saying that colour changes are gradual. Nonetheless, through applying these perceptive categories, I hope to have demonstrated their value in documenting prehistoric metalworking technology and skilfulness.

Through a *chaîne opératoire* approach updated with these perceptive categories, it is possible to show that different types of metals were indeed recognised and—to some extent—worked differently by prehistoric craftspeople. Axes made of the soft type I metal were hammer-hardened more forcefully in the EBA compared to LCA. This was the result of an increased standard in hardness and more risk needed to be taken with this material to achieve the standard, compared to type III metal. This highlights the role of risk in human-material relationships. Axes made of type I metal were still sometimes of inferior hardness, despite a skilful working. This is because the material simply did not allow a harder axe to be made of it, irrespective of the skill of the metalworker. Axes made of type III metal are typically good castings that resulted in axes with superior hardness values compared to the standard of the time, with little effort or skill. Axes made of type V metal typically are poor casts, again a ‘quality’ of the material, but this was recognised and many were worked attentively and with respect to the difficulties that they present, such as brittleness. A clear sign of skill.

In the EBA, at least five different types of metal were available and because of this wider array of raw material the craft of working metal had become less predictable and increasingly dependent on the attentiveness of the metalworker to recognise the differences between them. What is more, it is only through experiencing these different metals that knowledge about their differences could have been gained. From this, it is inferred that it would have been more difficult to become a skilled metalworker in this period than in the LCA, because of the increased variance of raw materials to work with. Furthermore, it is only in retrospect that certain materials (like tin-bronze) can be considered a logical progression. The quality that we perceive as ‘better’—in this case increased hardness—needed to be recognised and expressed first and its discovery is therefore fully dependent on skill.

I would like to draw three conclusions from the above observations.

Firstly: attention is drawn to the complex relationship between material, skill and quality. The craft perspective followed in this paper emphasises the role of the material. A high level of skill does not necessarily result in a high-quality object if the material does not afford it.

Secondly: certain raw materials influence the standard of the time and thus, in turn, how *other* raw materials are worked. This is especially interesting as one can envision how a similar interaction might have taken place between stone and bronze, or bronze and iron.

Thirdly: in the dialogue between material and maker, the material has the first word, because it provides us with qualities and potentials, as well as the last word, because it also sets the limitations of what can be done with it. It is *material that is the mother of innovation* and it is through skill that these innovations are brought about.

## Footnotes

1 My main three informants for hands-on knowledge on metalworking are Jeroen Zuiderwijk (based in the Netherlands), Holger Lönze (based in Ireland), and Neil Burridge (based in England). All three are experienced bronze casters with considerable knowledge of prehistoric metalwork.

2 A comparable methodological transcription is practiced to move from the environment to landscape, where the latter involves a qualitative categorisation (see Popa and Knitter 2016).

3 The idea that composition may be determined through colour measurement is even relevant for contemporary metallurgy (Kim et al. 2006)

4 If the composition contains both tin and a range of the other four elements and together these make up more than 5%, I have categorised the material as ‘Indet’ (Fig. 2)

5 I use the term ‘pure’ from a craft perspective. To the (archaeo)metallurgist, a copper with ~ 3% foreign elements would certainly not be considered pure.

6 In a patinated state, they turn green, which is likely why in China they refer to bronze as green copper (Cheng and Schwitter 1957, p. 355)

7 Heated metal can become hot-short, which means that it is under great thermal stress. Quenching or working it, or sometimes just a light tap, is enough to snap the metal at this stage. The result is a characteristic sharp and clean fracture.

8
*i*.*e*. weak/moderate shaping, annealing, and moderate/strong hardening.

## References

[CR1] Agricola G (1950). De Re Metallica.

[CR2] Agricola, G. (1955). *De Natura Fossilium (Textbook of Mineralogy)*. (M. C. Bandy & J. A. Bandy, Trans.). Geological Society of America. http://www.farlang.com/gemstones/agricola_textbook_of_mineralogy/page_002

[CR3] Apel J (2008). Knowledge, know-how and raw material—the production of late Neolithic Flint daggers in Scandinavia. Journal of Archaeological Method and Theory.

[CR4] Archbutt SL, Prytherch WE (1937). Effect of impurities in copper (Vol. 4).

[CR5] Berger, D. (2012). *Bronzezeitliche Färbetechniken an Metallobjekten nördlich der Alpen. Eine archäometallurgische Studie zur prähistorischen Anwendung von Tauschierung und Patinierung anhand von Artefakten und Experimenten*. Landesamt für Denkmalpflege und Archäologie Sachsen-Anhalt, Landesmuseum für Vorgeschichte.

[CR6] Biringuccio, V. (1990). *The Pirotechnia of Vannoccio Biringuccio. The Classic Sixteenth-Century Treatise on Metals and Metallurgy*. (C. S. Smith & M. T. Gnudi, Trans.). New York: Dover Publications.

[CR7] Bray, P. (2012). Before 29Cu became copper: tracing the recognition and invention of metalleity in Britain and Ireland during the third millennium BC. In M. J. Allen, J. Gardiner, & A. Sheridan (Eds.) *Is There a British Chalcolithic? People, Place and Polity in the later Third Millennium*.

[CR8] Bray PJ, Pollard AM (2012). A new interpretative approach to the chemistry of copper-alloy objects: Source, recycling and technology. Antiquity.

[CR9] Bray P, Cuénod A, Gosden C, Hommel P, Liu R, Pollard AM (2015). Form and flow: the “karmic cycle” of copper. Journal of Archaeological Science.

[CR10] Butler J, van der Waals JD (1964). Metal analysis, sam 1, and European prehistory. A review article. Helinium.

[CR11] Chase, W.T., 1994. Chinese Bronzes: Casting, Finishing, Patination, and Corrosion. In D.A. Scott, J. Podany & B.B. Considine (Eds.), *Ancient & Historic Metals: Conservation and Scientific Research: Proceedings of a Symposium Organized by the J. Paul Getty Museum and the Getty Conservation Institute, November 1991* (pp. 85–118). Marina del Rey, CA: Getty Conservation Institute.

[CR12] Cheng CF, Schwitter CM (1957). Nickel in Ancient Bronzes. American Journal of Archaeology.

[CR13] Childe VG (1963). The bronze age.

[CR14] Coghlan HH (1975). Notes on the prehistoric metallurgy of copper and bronze in the old world.

[CR15] Costin CL, Feinman GM, Price D (2001). Craft production systems. Archaeology at the millennium. A sourcebook.

[CR16] Devogelaere, J. (2017). The colour palette of antique bronzes: an experimental archaeology project. *EXARCH 2017/2*. https://exarc.net/issue-2017-2/ea/colour-palette-antique-bronzes-experimental-archaeology-project.

[CR17] Dobres, M. A. (2006). Skilled Production and Social Reproduction in prehistory and contemporary archaeology: a personal exegesis on dominant themes and their psychosocial influences. In J. Apel & K. Knutsson (Eds.), Skilled Production and Social Reproduction (Vol. 2, pp. 25–33). Uppsala: Societas Archaeologica Upsaliensis.

[CR18] Dobres MA (2010). Archaeologies of technology. Cambridge Journal of Economics.

[CR19] Earl B, Adriaens A (2000). Initial experiments on arsenical bronze production. Journal of the Minerals and Metals Society.

[CR20] Fang J-L, McDonnell G (2011). The colour of copper alloys. Historical Metallurgy.

[CR21] Frayling C (2011). On craftsmanship: Towards a new Bauhaus.

[CR22] Guettier, A. (1872). *A practical guide for the manufacture of metallic alloys: comprising their chemical and physical properties, with their preparation, composition, and uses.* (A. A. Fesquet, Trans.). Philadelphia: Henry Carey Baird, Industrial publisher.

[CR23] Hansen, S., 2013. Innovative metals: copper, gold and silver in the Black Sea Region and the Carpathian Basin During the 5th and 4th Millennium BC, in: S. Burmeister, S. Hansen, M. Kunst, & N.M. Müller-Scheessel (Eds.), *Metal Matters. Innovative Technologies and Social Change in Prehistory and Antiquity* (pp. 137–170). Rahden/Westf: Verlag Marie Leidorf.

[CR24] Harding AF (2000). European societies in the bronze age.

[CR25] Hiorns AH (1912). Mixed metals and metallic alloys.

[CR26] Hosler D (1995). Sound, color and meaning in the metallurgy of ancient West Mexico. World Archaeology.

[CR27] Hruby ZX, Flad RK, Bennett GP (2007). Rethinking craft specialization in complex societies: archaeological analyses of the social meaning of production.

[CR28] Hurcombe L (2007). A sense of materials and sensory perception in concepts of materiality. World Archaeology.

[CR29] Huxham J (1753). Medical and chemical observations upon antimony. Philosophical Transactions.

[CR30] Ingold T (2000). The perception of the environment. Essays in livelihood, dwelling and skill.

[CR31] Ingold T (2007). Materials against materiality. Archaeological Dialogues.

[CR32] Jones A (2002). Archaeological theory and scientific practice.

[CR33] Jones A (2004). Archaeometry and materiality: Materials-based analysis in theory and practice. Archaeometry.

[CR34] Jones A, MacGregor G (2002). Colouring the past: the significance of colour in archaeological research.

[CR35] Junk, M. J. (2003). *Material properties of copper alloys containing arsenic, antimony, and bismuth. The material of Early Bronze Age ingot torques.* Dissertation. Technischen Universität Bergakademie Freiberg.

[CR36] Kienlin TL (2006). Frühbronzezeitliche Randleistenbeile von Böhringen-Rickelshausen und Hindelwangen: Ergebnisse einer metallographischen Untersuchung. Praehistorische Zeitschrift.

[CR37] Kienlin TL (2007). Von den Schmieden der Beile: Zu Verbreitung und Angleichung metallurgischen Wissens im Verlauf der Frühbronzezeit. Praehistorische Zeitschrift.

[CR38] Kienlin TL (2008). Tradition and innovation in copper age metallurgy: Results of a metallographic examination of flat axes from eastern Central Europe and the Carpathian Basin. Proceedings of the Prehistoric Society.

[CR39] Kienlin TL (2008). Frühes Metall im Nordalpinen Raum. Eine Untersuchung zu technologischen und kognitiven Aspekten früher Metallurgie anhand der Gefüge frühbronzezeitlicher Beile.

[CR40] Kienlin TL (2010). Traditions and transformations: approaches to Eneolithic (copper age) and bronze age metalworking and society in eastern Central Europe and the Carpathian basin.

[CR41] Kienlin, T. L. (2013). Copper and bronze: Bronze Age metalworking in context. In H. Fokkens & A. Harding (Eds.), The Oxford Handbook of the European Bronze Age (pp. 414–436). Oxford: Oxford University Press. http://www.oxfordhandbooks.com/view/10.1093/oxfordhb/9780199572861.001.0001/oxfordhb-9780199572861-e-23. Accessed 8 November 2016.

[CR42] Kienlin TL, Bischoff E, Opielka H (2006). Copper and bronze during the Eneolithic and early bronze age: A metallographic examination of axes from the Northalpine region. Archaeometry.

[CR43] Killick D (2004). Social constructionist approaches to the study of technology. World Archaeology.

[CR44] Killick D, Fenn T (2012). Archaeometallurgy: the study of preindustrial mining and metallurgy. Annual Review of Anthropology.

[CR45] Kim CW, Kim HG, Suk HG (2006). A study on the composition determination of cu alloys by image processing technology. Solid State Phenomena.

[CR46] Kristiansen K, Larsson B (2005). The rise of bronze age society. Travels, transmissions and transformations.

[CR47] Kuijpers, M. H. G. (2017) The Bronze Age, a World of Specialists? Metalworking from the Perspective of Skill and Material Specialization. *European Journal of Archaeology*, 1–22. 10.1017/eaa.2017.59.

[CR48] Kuijpers MHG, Kienlin TL, Zimmermann A (2012). Towards a deeper understanding of metalworking technology. Beyond elites. Alternatives to hierarchical Systems in Modelling Social Formations. International conference at the Ruhr-Universität Bochum, Germany, October 22–24, 2009.

[CR49] Kuijpers MHG, Sørensen M-LS, Rebay-Salisbury K (2013). The sound of fire, taste of copper, feel of bronze, and colours of the cast: sensory aspects of metalworking technology. Embodied knowledge: Historical perspectives on belief and technology.

[CR50] Kuijpers, M. H. G. (2015). Some thoughts on quality and skill in Early Bronze Age axes. In E. A. G. Ball & S. Arnoldussen (Eds.), *Metaaltijden 2. Bijdragen in de studie van de metaaltijden.* (Vol. 2, pp. 19–29). Sidestone Press.

[CR51] Kuijpers, M. H. G. (2018). *An Archaeology of Skill: Metalworking Skill and Material Specialization in Early Bronze Central Europe*. London: Routledge.

[CR52] Lechtman H (1996). Arsenic bronze: Dirty copper or chosen alloy? A view from the Americas. Journal of Field Archaeology.

[CR53] Lechtman H, Klein S (1999). The production of copper-arsenic alloys (arsenic bronze) by Cosmelting: modern experiment, ancient practice. Journal of Archaeological Science.

[CR54] Leusch, V., Armbruster, B., Pernicka E., & Slavčev, V. (2015). On the invention of gold metallurgy: the gold objects from the Varna I cemetery (Bulgaria)—technological consequence and inventive creativity. *Cambridge Archaeological Journal, 25*(1), 353–376. 10.1017/S0959774314001140.

[CR55] Makar HV, Riley WD (1985). Metallurgical effects of impurities in recycled copper alloys.

[CR56] Martinón-Torres, M. & Killick, D. (2015). Archaeological Theories and Archaeological Sciences, in: A. Gardner, M. Lake, U. Sommer (Eds.), *The Oxford Handbook of Archaeological Theory*. Oxford University Press. http://www.oxfordhandbooks.com/10.1093/oxfordhb/9780199567942.001.0001/oxfordhb-9780199567942-e-004

[CR57] McCreight T (2010). Complete metalsmith.

[CR58] McKerrell H, Tylecote RF (1972). The working of copper-arsenic alloys in the early bronze age and the effect on the determination of provenance. Proceedings of the Prehistoric Society.

[CR59] Merkl M (2010). Bell beaker metallurgy and the emergence of Fahlore-copper use in Central Europe. Interdisciplinaria Archaeologica. Natural Sciences in Archaeology.

[CR60] Mödlinger M, Sabatini B (2016). A re-evaluation of inverse segregation in prehistoric As-Cu objects. Journal of Archaeological Science.

[CR61] Mödlinger, M., de Oro Calderon, R & Haubner, R. 2017a. Arsenic loss during metallurgical processing of arsenical bronze. *Archaeological and Anthropological Sciences*: p.1–8. doi: 10.1007/s12520-017-0534-1

[CR62] Mödlinger, M., Kuijpers, M.H.G., Braekmans, D. & Berger, D. (2017b). Quantitative comparisons of the color of CuAs, CuSn, CuNi, and CuSb alloys. *Journal of Archaeological Science.*

[CR63] Mordant C, Pernot M, Rychner V (1998). Les Analyses de composition du métal: Leur apport à l’archéologie de l’âge du bronze.

[CR64] Nerantzis N (2012). Shaping bronze by heat and hammer: an experimental reproduction of Minoan copper alloy forming techniques. Mediterranean Archaeology and Archaeometry.

[CR65] Northover JP, Hauptman A, Pernicka E, Wagner GA (1989). Properties and use of arsenic-copper alloys. Archäometallurgie der Alten Welt.

[CR66] Olausson, D., (2017). Knapping skill and craft specialization in Late Neolithic Flint Daggers. Lithic Technology, 1–13. doi:10.1080/01977261.2017.1364328

[CR67] Ottaway BS (1994). Prähistorische Archäometallurgie.

[CR68] Ottaway, B. S. & Roberts, B. W. (2008). The Emergence of Metalworking. In A. Jones (Ed.), *Prehistoric Europe: Theory and Practice*. London: Blackwell.

[CR69] Pare CFE, Pare CFE (2000). Bronze and the bronze age. Metals make the world go round. The supply and circulation of metals in bronze age Europe.

[CR70] Pearce M (2007). Bright blades and red metal. Essays on north Italian prehistoric metalwork (Vol. 14).

[CR71] Pollard AM, Bray P (2007). A bicycle made for two? The integration of scientific techniques into archaeological interpretation. Annual Review of Anthropology.

[CR72] Popa, C. N., & Knitter, D. (2016). From environment to landscape. Reconstructing environment perception using numerical data. *Journal of Archaeological Method and Theory, 23*(4), 1285–1306. 10.1007/s10816-015-9264-9 PMC5750687 29368750

[CR73] Pye D (1995). The nature and art of workmanship.

[CR74] Radivojević M, Rehren T, Kuzmanović-Cvetković J, Jovanović M, Northover JP (2013). Tainted ores and the rise of tin bronzes in Eurasia, c. 6500 years ago. Antiquity.

[CR75] Roberts BW, Thornton CP (2014). Archaeometallurgy in global perspective: methods and syntheses.

[CR76] Rowlands M, Bintliff JL (1984). Conceptualizing the European bronze and early iron age. European social evolution: archaeological perspectives.

[CR77] Scott DA (2011). Ancient metals: microstructure and metallurgy.

[CR78] Smith CS (1975). Metallurgy as a human experience. Metallurgical Transactions A.

[CR79] Sofaer, J. R. (2010). Technology and Craft. In T. Earle & K. Kristiansen (Eds.), *Organizing Bronze Age Societies. The Mediterranean, Central Europe, and Scandinavia compared* (pp. 185–217). Cambridge University Press.

[CR80] Thornton CP, Roberts BW, Kienlin TL (2009). Archaeometallurgy: evidence of a paradigm shift?. Metals and societies. Studies in honour of Barbara S. Ottaway.

[CR81] Untracht O (1969). Metal techniques for craftsmen: a basic manual for craftsmen on the methods of forming and decorating metals (reprinted 2010).

[CR82] Vandkilde, H. (2010). Metallurgy, Inequality and Globalization in the Bronze Age - discussant’s commentary on the papers in the metallurgy session. In H. Meller & F. Bertemes (Eds.), *Der Griff nach den Sternen. Wie Europas Eliten zu Macht und Reichtum kamen. Internationales Symposium in Halle (Saale) 16.-21. Februar 2005* (Vol. II, pp. 903–910). Halle (Saale): Landesamt für Denkmalpflege und Archäologie Sachsen-Anhalt & Landesmuseum für Vorgeschichte.

[CR83] Villegas MAU, Martinón-Torres M-T (2012). Composition, colour and context in Muisca votive metalwork (Colombia, AD 600-1800). Antiquity.

[CR84] Wang Q, Ottaway BS (2004). Casting experiments and microstructure of archaeologically relevant bronzes.

